# The Role of Radiological Imaging in the Diagnosis and Treatment of Urolithiasis: A Narrative Review

**DOI:** 10.7759/cureus.33041

**Published:** 2022-12-28

**Authors:** Moneera M Al-Shawi, Noor A Aljama, Rajeh Aljedani, Mohammed H Alsaleh, Nawaf Atyia, Ali Alsedrah, Mazen Albardi

**Affiliations:** 1 Family Medicine, King Fahad University Hospital, Imam Abdulrahman Bin Faisal University, Dammam, SAU; 2 Medicine and Surgery, Imam Abdulrahman Bin Faisal University, Al-Qatif, SAU; 3 Medicine, King Abdulaziz University Hospital, Jeddah, SAU; 4 Medicine, Faculty of Medicine, Jagiellonian University, Krakow, POL

**Keywords:** urolithiasis prognosis, treatment of urolithiasis, radiological imaging modality, urolithiasis clinical presentation, urolithiasis epidemiology

## Abstract

The incidence and prevalence of urolithiasis have been increasing worldwide for the last several decades. This increase could be attributed to many factors, including missed cases of small stones, a sedentary lifestyle, a high BMI, a lack of physical activity, and poor dietary intake. In addition, the increased incidence of co-morbidities such as diabetes, dyslipidemia, infections, and multiple urinary tract surgeries could contribute significantly to the formation of urolithiasis. Radiology has a major role in diagnosing a variety of these stone types and can be used in planning management approaches, either as guidance or as a direct therapeutic method for stones. Because of the availability, safety, cost, and effectiveness of radiological imaging nowadays, urolithiasis is rarely missed; furthermore, the availability of radiological treatment options decreases the need for surgical intervention for urolithiasis, which minimizes hospital stay and surgical-related complications.

This review aims to scope and analyze the role of radiological imaging modalities in reaching a diagnosis and planning treatment options for urolithiasis in different circumstances. Information was gathered from relevant peer-reviewed publications in PubMed and thereafter refined and summarized to provide a comprehensive review. The selected indexing terms included "radiological imaging modality," "treatment of urolithiasis," and "diagnosis of urolithiasis," among others.

## Introduction and background

Urolithiasis has been a problem for clinicians since Hippocrates' time. It is a common problem with increasing prevalence worldwide and affects a wide spectrum of the population. Due to a sedentary lifestyle, elevated BMI, especially in those who exceed 30 kg/m^2^, nutritional intake, and a lack of physical exercise, urinary stone disease has become more prevalent over the past few decades in both developed and developing countries [1-3]. It is more common in countries with hot climates and among white people. The peak age in males is 40-60 years; in females, it is 30 years. The pediatric age group has an incidence rate of up to 3%. Urinary tract stones are mainly categorized based on composition into calcium, struvite, and uric acid; other uncommon types of stones include those that develop with indinavir use for the treatment of HIV, brushite, cysteine, and protein matrix [4]. Recent studies have also demonstrated a changing composition of urolithiasis as well as an increase in the incidence of stone disease in females and younger patients over the last decade [2]. A large cohort study at the University of Wisconsin showed the prevalence of urolithiasis in the asymptomatic population is almost 8%, with most of the cases associated with obesity, diabetes, and old age, even if there is no significant correlation between these conditions and asymptomatic urolithiasis [5,6].

As the most common site for the development of urolithiasis is at the distal nephron near the urinary papilla [5], the leading symptom is suddenly occurring colicky pain in the flank radiating to the hypochondrium, one of the exceptions is patients with staghorn calculi, they could only experience mild lumbar pain. The pain tends to be excruciating and varies according to the patient's position and the location of the stone. The pain is not relieved by postural changes or non-narcotic medications and can be associated with nausea and vomiting [1,7]. Cohort studies initiated at the University of Wisconsin showed that most of the stones were located on the right side, less on the left side, and the lowest percentage was bilateral kidney stones [6]. Some renal stones remain asymptomatic if they are small enough to pass through the ureter; others may present with septic features from obstructive pyelonephritis [3]. Other conditions, depending on the presentation, have similar clinical features to urolithiasis; the differential diagnoses include those of the acute abdomen such as pyelonephritis, appendicitis, pancreatitis, and cholecystitis. Also, pain originating from ovarian pathology, cardiac or musculoskeletal sources, and pleuritic chest pain may have similar presentations to urolithiasis [7].

The major prognostic factors are stone size and location. The likelihood that a ureteral stone will pass appears to be determined by its size; stones less than 5 mm in size should be given an opportunity to be conservatively managed. As stones increase in size by more than 4 mm, the urological referral becomes crucial. However, referral to a urologist is indicated for patients with a stone greater than 5 mm in size and for patients with a ureteral stone that has not passed after two to four weeks of observation [1,5]. Furthermore, the location of the stone impacts the management outcome, with the treatment success rates being better for calculi located in the lower third of the ureter [2]. Appropriate management of urolithiasis has important clinical implications due to its association with complications such as infection, chronic kidney disease, and a high rate of recurrence [1,5]. Patients who are not treated may experience relapse within 5-10 years [5].

## Review

Diagnostic tools

Imaging plays a critical role in the initial diagnosis, follow-up, and urological management of urinary tract stone disease. Historically, urologists have used a variety of imaging modalities, including plain radiography of the kidneys, ureters, and bladder (KUB), intravenous pyelogram (IVP), ultrasound (US), magnetic resonance urography (MRU), and computed tomography (CT), each with its advantages and limitations. Until recently, IVP was considered the gold standard for diagnosing renal calculi, but this modality has largely been replaced by non-contrast CT (NCCT) due to its high sensitivity and specificity of 99% and the ease of performing the study. In addition, it can show indirect or secondary signs of obstruction such as periureteral fluid collection, perihepatic fat stranding, or hydronephrosis [3,5,6,8-11].

Computed tomography

Non-contrast CT (NCCT) offers several advantages compared with alternative imaging techniques, such as plain radiography and US, including high sensitivity and specificity (>95% and >96%, respectively) for the detection of stones, easy availability, faster acquisition, and the absence of administration of intravenous contrast [2]. It can approximately detect all stones regardless of their location, size, and composition, but the main exception is indinavir stones and pure matrix protein that cannot be detected in CT; these stones form in patients treated for immune deficiency virus (HIV) [10]. One of the advantages of CT is that it can permit the diagnosis of secondary signs of obstruction presenting as hydroureteronephrosis or perinephric edema [4]. Also, CT can assess congenital conditions of the urinary tract and renal or urothelial neoplasms [2]. Furthermore, due to its ability to visualize abdominal structures more accurately than other modalities, CT is reliable in rolling out differentials of acute abdomen such as appendicitis and tubo-ovarian pathology [4]. A study of 1500 CT scans for the evaluation of acute flank pain was conducted, and additional conditions (68% of cases) in addition to urolithiasis were reported. Despite these advantages, the radiation risk is concerning, especially in young patients and those with recurrent diseases [12]. Dual-energy computed tomography (DECT) has the unique ability to differentiate stone composition, which aids in predicting the efficacy of extracorporeal shock wave lithotripsy (ESWL) in various stone types. CT remains the gold standard method to detect residual stone fragments and assess post-treatment complications appropriately [4]. Contrast-enhanced computed tomography can visualize a variety of other conditions that may be missed or incompletely characterized by stone protocol CT and differentiate between distal ureteral calculus from phlebolith. Furthermore, CT with contrast has a high sensitivity for renal calculi (3 mm). Incorporating stone-specific clinical parameters, such as urinalysis confirming hematuria and urolithiasis history, allows patients to be triaged to either stone protocol CT or routine contrast-enhanced CT [4,13,14].

Ultrasonography

The renal collecting systems, renal parenchyma, and bladder may all be evaluated quite well with ultrasound (US), but the ureters are difficult to visualize, especially in patients with a lot of intestinal gas or subcutaneous fat [4]. The US is a helpful initial imaging technique, especially for those who are frequently affected by stones and are at high risk for radiation exposure such as children and pregnant women. Reduced sensitivity and specificity compared to a CT scan are the main disadvantages of the US for the diagnosis of urolithiasis. Other limitations of ultrasonography include the inaccuracy of stone size estimation and poor detection in patients with a high BMI [15]. Although less precise than CT, US and plain radiography can be used to exclude conditions like obstructive hydronephrosis and large recurring radiopaque urinary tract calculi, respectively [4]. A study conducted in the National Hospital Ambulatory Medical Care Survey (NHAMCS) between 1996 and 2007 states that, despite increasing utilization of CT in hospitals, it showed no effects on the number of hospitalizations or management of the patients [16]. Point-of-care ultrasound (POCUS) has moderate accuracy in making the diagnosis of urolithiasis. Nevertheless, it may be safely used as first-line imaging in emergency department patients with suspected symptomatic urolithiasis [17]. US has a big role in detecting stones and should be the initial imaging in acute stone episodes as it can be done bedside and is free from ionizing radiation. It can demonstrate hyperechogenicity accompanied by a posterior shadow. However, the presence of hydronephrosis is highly suggestive of a large stone (> 5 mm) in those presenting with renal colic [18]. When patients with distal ureteral stones are detected and given conservative therapy, the US has been found to be a good substitute for CT in follow-up imaging. A prospective study looked at individuals whose initial CT revealed a distal ureteral stone and whose subsequent imaging included US in addition to a CT, radiograph, or both. They discovered that the US has a high specificity of 99.1% and sensitivity of 94.3% for finding lingering stones. In comparison to CT, ultrasonography, when combined with KUB, provides adequate sensitivity and specificity with less radiation [12].

Magnetic resonance urography

Magnetic resonance urography (MRU) is not commonly used in urolithiasis, but it could be used as an effective alternative method in patients who need to avoid radiation exposure, especially in cases of pregnancy, children, or renal impairment to avoid the administration of contrast. MRU gives us the advantage of showing comprehensive, detailed anatomy regarding the urinary tract and its related soft tissue, therefore helping in the identification and management of calculi. Moreover, in pregnant women, MRU can be useful to differentiate physiological from pathological ureteral obstruction [12,19]. In research comparing the effectiveness of MRI and CT in individuals with acute ureteric blockage, perirenal fluid was visible on MRI with a sensitivity that was higher (77%) than the CT-indicated stranding (45%). On MRI, the presence of fluid and ureteric dilatation was associated with 93% sensitivity (CT 80%), 95% specificity (CT 85%), and 94% accuracy (CT 81%). Another unique advantage of MRU is detecting indinavir-induced stones in patients with HIV, which are radiolucent stones in CT [12].

Plain radiograph

Plain radiography of the kidney-ureter-bladder (KUB) was commonly used to diagnose urolithiasis, as most stone types were calcium stones, which could be seen in the KUB [12]. Nowadays it is not a favorable method for diagnosing urolithiasis. This is due to the fact that the sensitivity of KUB in detecting stones is limited (45-58%), as some types of stones are radiolucent, making it difficult to distinguish them from the bowel content overlying them. On the other hand, KUB is more favorable to be used during pretreatment sitting in patients planned for ESWL, and postoperatively for follow-up evaluations and ureteral stent placement [4,12]. KUB was reported to have a sensitivity of 96% and a specificity of 91% for diagnosing ureteral stones when combined with the US [12].

Intravenous pyelogram

Historically, the IVP was the most widely used imaging tool in diagnosing urolithiasis, but as science advanced, the CT became superior to the IVP due to the benefits shown by the CT. According to a prospective study comparing these two modalities, the CT showed a sensitivity of 94.1 and specificity of 94.2, whereas the IVP showed a sensitivity of 85.2 and specificity of 90.4. Furthermore, NCCT had the advantages of being faster, safer due to the absence of contrast use, and more cost-effective than IVP. Despite its limited role nowadays, IVP could be used in certain conditions where anatomic identification is challenging [12]. In selected cases during pregnancy, it showed that when the US reveals no findings for urolithiasis, IVP can be used as an adjunctive method with extreme safety precautions as well as when the benefits outgain radiation exposure to the fetus. Studies showed that IVP was superior to the US in confirming urolithiasis during pregnancy, with a percentage of 96% and 60% for IVP and the US, respectively [19].

Management

To begin the management, the size and location of the stones are used as markers. Conservative medical expulsive therapy (MET) management, including alpha-blockers and calcium channel blockers, could be initiated in asymptomatic people with small stones (8 mm); they facilitate the passage of ureteral stones and play a role in relieving ureteral muscle spasms [5]. On the other hand, the emergence of increasingly effective and minimally invasive techniques such as ESWL and ureteroscopy (URS) has contributed to the gradual disappearance of open surgical procedures to treat ureteric stones, renal stones (20 mm), or lower renal pole stones (10 mm) [5,20,21]. Furthermore, renal stones larger than 2 mm or lower renal pole stones smaller than 1 mm may be treated with percutaneous nephrolithotomy (PCNL) [5,22,23].

Extracorporeal shock wave lithotripsy

ESWL has been introduced into clinical practice since 1980 [21,24]. It is a minimally invasive method of treating urinary stones that has a success rate of 70-80% after the procedure. It is currently considered one of the most recommended treatment options for small- and medium-sized stones (renal stones less than 20 mm or lower renal pole stones less than 10 mm) in most guidelines internationally [21,25]. The use of medical expulsive therapy in the form of alpha-blockers, especially tamsulosin, has been proven by many randomized control trials and several meta-analyses that support the use of tamsulosin after ESWL to facilitate the passage of ureteral stones [26]. It has been shown that ESWL has fewer complications than other modalities, with no need for anesthesia and a shorter hospital stay. Unfortunately, it has a higher recurrence rate compared to ureteroscopy [27,28]. Moreover, despite new modalities, ESWL remains the patient's first option in many circumstances [28,29].

Ureterorenoscopy

Flexible ureteroscopes have a beneficial role in treating renal stones endoscopically through natural orifices. Many analyzed reports from several randomized controlled trials have shown that ureteroscopy achieved high success rates in managing ureteric stones and provided a better stone-free rate after treatment, fewer re-interventions, fewer sick leave days, and lower healthcare costs. However, compared to ESWL and PCNL patients, those who had undergone ureteroscopy, unfortunately, continue to have remnant stone fragments that may cause recurrent symptoms, obstruction, infection, and a longer hospital stay [30,31].

Percutaneous nephrolithotomy

PCNL was developed by Fernström and Johansson and introduced in 1976. It has been used instead of open surgery to treat renal stones larger than 2 mm or lower pole stones larger than 1 mm [4]. PCNL is considered the treatment of choice for staghorn calculi, with a higher stone-free rate of 85% to 93%. It is contraindicated in pregnancy, bleeding disorders, and uncontrolled urinary tract infections [32].

Fluoroscopy

Fluoroscopy has been the main imaging modality since it was introduced in clinical practice in 1981. Its main goal is to guide percutaneous access to the collecting system. Unfortunately, it has been associated with disadvantages such as limited visualization of the visceral organs around the kidney. Additionally, the accompanying radiation exposure to the patient, attending physician, and medical staff in the operating room has always been a possible risk factor for adverse effects [18,30].

Fluoroscopy-free retrograde intrarenal surgery

Fluoroscopy-free retrograde intrarenal surgery seems to be technically feasible and safe for the treatment of renal and proximal ureteral stone disease in uncomplicated, selected cases [24]. A study discussed the feasibility of fluoroscopy-free retrograde intrarenal surgery and the efficacy of such treatment. A total of 93 patients underwent the procedure, with a stone-free rate of 69.9%; residual fragments and residual stones were detected at 13.9% and 16.1%, respectively; and no major complications were reported [32]. Another study discussed the same context regarding the efficacy of fluoroscopy-free retrograde intrarenal surgery; the stone-free rate reached 90.37%. This percentage was derived from 122 of 135 renal stone patients [33].

## Conclusions

The rise in urolithiasis incidence and changes in its composition in recent decades prompted researchers to pay closer attention to the effectiveness of imaging modalities in diagnosing stones. Due to its higher sensitivity and specificity in diagnosing stones, time and cost-effectiveness, and high advantage in leading management plans and follow-up for complications, CT scans have remained the gold standard imaging modality until today. However, the radiation risk should not be underestimated, especially for those who frequently suffer from stones, those who are pregnant, or children. As an alternative approach, we encourage the use of other imaging modalities initially in patients with a high risk of radiation exposure. Combining KUB with US showed adequate efficacy in detecting ureteral stones and helped reduce radiation and cost. Furthermore, bedside US has a role in an emergency setting to rule out obstructive hydronephrosis in patients suspected of urolithiasis. As radiologists move toward reducing radiation exposure from CT and fluoroscopy; US technology seems to have a promising role in the future of medicine and radiology, this can be attributed to its significant advances over the last several decades, and this trend is likely to continue, making the US even more accessible in the future. Currently, ESWL, URS, and PCNL have developed significantly and are efficient and reliable. We urge and recommend any society that is interested in urinary stones to develop a scoring system that helps in diagnosing urinary stones and thus helps in speedy diagnosis, the avoidance of resource waste, and the application of imaging modalities to predict the response of urinary stones to a specific treatment.
